# Establishment of a Simple and Efficient Reverse Genetics System for Canine Adenoviruses Using Bacterial Artificial Chromosomes

**DOI:** 10.3390/v12070767

**Published:** 2020-07-16

**Authors:** Hiromichi Matsugo, Tomoya Kobayashi-Kitamura, Haruhiko Kamiki, Hiroho Ishida, Akiko Takenaka-Uema, Shin Murakami, Taisuke Horimoto

**Affiliations:** Department of Veterinary Microbiology, Graduate School of Agricultural and Life Sciences, The University of Tokyo, 1-1-1 Yayoi, Bunkyo-ku, Tokyo 113-8657, Japan; matsugo-hiromichi328@g.ecc.u-tokyo.ac.jp (H.M.); t.kitamura@affrc.go.jp (T.K.-K.); kamiki-haruhiko295@g.ecc.u-tokyo.ac.jp (H.K.); ishida-hiroho417@g.ecc.u-tokyo.ac.jp (H.I.); atakiko@mail.ecc.u-tokyo.ac.jp (A.T.-U.); amurakam@mail.ecc.u-tokyo.ac.jp (S.M.)

**Keywords:** canine, adenovirus, reverse genetics, vector, bacterial artificial chromosome

## Abstract

Canine adenoviruses (CAdVs) are divided into pathotypes CAdV1 and CAdV2, which cause infectious hepatitis and laryngotracheitis in canid animals, respectively. They can be the backbones of viral vectors that could be applied in recombinant vaccines or for gene transfer in dogs and in serologically naïve humans. Although conventional plasmid-based reverse genetics systems can be used to construct CAdV vectors, their large genome size creates technical difficulties in gene cloning and manipulation. In this study, we established an improved reverse genetics system for CAdVs using bacterial artificial chromosomes (BACs), in which genetic modifications can be efficiently and simply made through BAC recombineering. To validate the utility of this system, we used it to generate CAdV2 with the early region 1 gene deleted. This mutant was robustly generated and attenuated in cell culture. The results suggest that our established BAC-based reverse genetics system for CAdVs would be a useful and powerful tool for basic and advanced practical studies with these viruses.

## 1. Introduction

Canine adenovirus (CAdV), a double-stranded DNA virus that belongs to the genus *Mastadenovirus* in the family *Adenoviridae*, is divided into pathotypes CAdV1 and CAdV2, the former of which causes infectious hepatitis and encephalitis in dogs, bears, red foxes, and wolves [1,2,3], and the latter causes infectious laryngotracheitis in dogs [4]. The molecular basis of this pathogenic difference remains unknown. Outbreaks of CAdV infections in wildlife and animal shelters have been reported in recent years [5,6]. To protect domestic dogs from infections by this virus, attenuated CAdV2 vaccines, which are also effective against serologically cross-reactive CAdV1 infections, are widely used.

Currently, several adenovirus vectors are being commonly employed and tested for clinical use. The replication-defective constructs, in which essential genes (e.g., early region 1 (*E1*)) are replaced with foreign therapeutic or protective antigen genes, can be used for gene and cancer therapies and as recombinant vaccines, whereas the replication-competent constructs, which preferentially replicate in cancer cells only, can be applied for oncolytic therapy [7,8]. Several human adenovirus (HAdV)-based vectors for gene transfer have been evaluated for clinical use in humans for many years. However, any pre-existing cellular or humoral immunity against HAdV can prevent efficient gene transfer and affect the duration of gene expression. By contrast, humans do not have any pre-existing immunity against CAdVs [9], which presents CAdV-based vectors as promising for human use. Additionally, CAdV can potentially be an oncolytic virus for canine cancers [10].

The reverse genetics system, through which an intended gene manipulation can be introduced into the viral genome, is a powerful tool for use in basic studies on CAdVs (e.g., on their pathogenesis) as well as in practical studies (e.g., on CAdV-based vectors and recombinant vaccines). Several techniques to construct recombinant AdVs, including CAdVs, have been reported. In vitro ligation or its improved method, in which foreign DNA is directly ligated to AdV DNA digested with restriction enzyme, requires a unique restriction enzyme site and has low ligation efficiency [11,12,13,14]. The mammalian cell-based homologous recombination method, in which foreign DNA flanked by homology arms and adenovirus DNA are introduced into cells, requires long homology arms and repeated plaque purifications [15,16]. The bacteria-based homologous recombination method, in which the full-length AdV genome is cloned into plasmid via homologous recombination in the *Escherichia coli* BJ5183 strain, is widely used to construct infectious clones and to manipulate viral DNA. However, this method also requires long homology arms and a unique restriction enzyme site [9,17,18,19,20,21]. The yeast artificial chromosome-based method, which requires an additional yeast host, is infrequently used [22].

To circumvent these issues, in this study, we established a bacterial artificial chromosome (BAC)-based reverse genetics system for CAdV, in which genetic modifications could be easily made through BAC recombineering, which requires only short homology arms [23]. Additionally, we generated an E1-deleted CAdV2 mutant to validate the utility of this BAC-based reverse genetics system. This mutant would be a promising backbone for the construction of viral vectors.

## 2. Materials and Methods

### 2.1. Cells and Viruses

Madin–Darby canine kidney (MDCK) cells were obtained from the American Type Culture Collection (CCL-34). These and the MDCK-CAdV2-E1-26 cells (described in Section 2.6) were maintained in Dulbecco’s modified Eagle’s medium (DMEM) supplemented with 5% fetal bovine serum (FBS), penicillin (100 U/mL), and streptomycin (0.1 mg/mL) at 37 °C in 5% CO_2_. CAdV1 (strain D43 [24]) and CAdV2 (strain A2 [25]) were propagated in MDCK cells in DMEM supplemented with 1% FBS. CAdV2-ΔE1-Venus (described in Section 2.6) was propagated in MDCK-CAdV2-E1-26 cells.

### 2.2. Next-Generation Sequencing

Viral DNA was extracted from 50 µL of the supernatant of CAdV1- or CAdV2-infected cell cultures using a QIAamp DNA mini kit (QIAGEN, Tokyo, Japan). The DNA was subjected to next-generation sequencing using the Ion Personal Genome Machine System (Thermo Fisher Scientific, Tokyo, Japan) according to the manufacturer’s protocol. The sequences were arranged in order by mapping them to CAdV1 (GenBank accession nos. AC_000003 and NC_001734) and CAdV2 (GenBank accession no. U77082) reference sequences using the CLC Genomic Workbench (version 8; CLC Bio, Aarhus, Denmark), and then deposited in the DNA Data Bank of Japan (DDBJ), GenBank, and European Molecular Biology Laboratory (EMBL) databases (Accession Nos.: CAdV1 D43 strain, LC557010; CAdV2 A2 strain, LC557011).

### 2.3. Cloning of the CAdV Genome into a Bacterial Artificial Chromosome Vector

The primers used in this study are listed in Supplementary Table S1. To clone the galactokinase (*galK*) and kanamycin resistance (*kan*^R^; hereinafter designated *Kn*) genes under the same promoter, the polymerase chain reaction (PCR) was applied to amplify the *galK* expression cassette from pGalK (National Cancer Institute) using the primers pUC19-GalK F, Kn-GalK R, and *Kn* from pCR-Blunt II-TOPO (Invitrogen/Thermo Fisher Scientific, Tokyo, Japan) using the primers GalK-Kn F and pUC19-Kn R. Both PCR products were ligated into HindIII-XbaI-digested linearized pUC19. The resultant plasmid was named pGalK-Kn. To add 50-bp homology arms and restriction enzyme sites to the *galK* expression cassette, the cassette was amplified from pGalK using the primers CAdV1-ITR-GalK F1 and CAdV1-ITR-GalK R1 or CAdV2-ITR-GalK F1 and CAdV2-ITR-GalK R1. Then, the PCR products were amplified using the primers CAdV1-ITR FR2 or CAdV2-ITR FR2. The resultant fragments were digested with BamHI and cloned into BamHI-digested pSMART BAC using the CopyRight v2.0 BAC Cloning Kit (Lucigen, Madison, WI, USA). Then, the plasmids were used to transform BAC-Optimized Replicator v2.0 Electrocompetent cells (Lucigen) using a MicroPulser (BioRad, Hercules, CA, USA) at preset condition Ec1. The resultant BAC vectors were named pBAC-CAdV1-ITR-GalK and pBAC-CAdV2-ITR-GalK.

To clone the full-length CAdV1 and CAdV2 genomes, we used *Escherichia coli* strain SW102 (National Cancer Institute), which contains a fully functional galactose operon (except with *galK* deleted) and in which Red recombinase can be induced at 42 °C [23]. In brief, SW102 cells containing pBAC-CAdV1-ITR-GalK or pBAC-CAdV2-ITR-GalK were incubated overnight in 1 mL of Luria–Bertani (LB) medium supplemented with 12.5 µg/mL of chloramphenicol (CP) at 32 °C with shaking at 180 rpm. On the following day, 0.5 mL of the overnight culture was diluted in 10 mL of CP-containing LB medium and incubated at 32 °C for 1.5–2 h until the OD_600_ reached 0.4–0.6. Then, Red recombinase was induced through heat shock at 42 °C for 15 min. Thereafter, the cells were chilled on ice for 10–15 min and then washed three times with cold H_2_O. The cell pellet was resuspended in 30–50 µL of cold H_2_O and kept on ice. A 25-µL aliquot of the cells was mixed with 1 µg of CAdV1 DNA or 500 ng of CAdV2 DNA, and the mixture was electroporated using a MicroPulser (BioRad) at preset condition Ec1. After the electroporation, the cells were recovered in CP-containing LB medium at 32 °C for 4.5 h. Then, the cells were washed twice with M9 medium and plated onto M63 minimum medium plates supplemented with glycerol, leucine, biotin, 2-deoxygalactose, and CP. These plates were incubated at 32 °C for 3 days, following which the bacterial colonies were screened by PCR using the following primers: SL1 and CAdV1 259 R, SL1 and CAdV1 30090 F, pSMART BAC 159 R and CAdV1 259 R, and pSMART BAC 159 R and CAdV1 30090 F for CAdV1; and SL1 and CAdV2 300 R, SL1 and CAdV2 30880 F, pSMART BAC 159 R and CAdV2 300 R, and pSMART BAC 159 R and CAdV2 30880 F for CAdV2. Then, the positive BACs were digested with restriction enzymes, and their sizes were confirmed by electrophoresis. The resultant BACs were named pBAC-CAdV1 and pBAC-CAdV2.

### 2.4. Generation of Recombinant CAdVs

To generate recombinant CAdVs, the respective BACs were digested with PvuI. Thereafter, the linearized viral genomic DNA was transfected into MDCK cells using polyethylenimine (Polysciences, Warrington, PA, USA) according to the manufacturer’s instructions. At 5–6 days post-transfection, the medium and the cells were frozen and thawed three times, and the supernatants obtained from centrifugation of the cell cultures were collected and used to infect MDCK cells. Once 50–80% of the cells showed cytopathic effects, the culture supernatants were collected and stored at −80 °C. The genomic DNA was extracted from the recombinant wild-type (rWT) CAdVs, and the restriction patterns were compared with those of the genomic DNA from wild-type (WT) CAdVs.

### 2.5. Modification of the CAdV Genome in the Bacterial Artificial Chromosome Vector

To construct the CAdV2 infectious clone with the *E1* region deleted, *galK-Kn* cassettes were amplified from pGalK-Kn using the primers CAdV2-E1-GalK-Kn F and CAdV2-E1-GalK-Kn R. SW102 cells containing pBAC-CAdV2 were heat shocked to induce Red recombinase, following which they were electroporated with 100 ng of the PCR product. Then, the recovered cells were plated on LB medium plates containing CP and 25 µg/mL kanamycin. After 18–24 h, the bacterial colonies were passaged to MacConkey medium plates supplemented with CP and d-galactose. Then, the red bacterial colonies that formed on the plates, which were GalK-positive SW102 cells, were transferred to LB medium containing CP and kanamycin and incubated overnight. On the following day, 0.5 mL of the overnight culture was diluted in 10 mL of CP-containing LB medium and incubated at 32 °C for 1.5–2 h; then, Red recombinase was induced by heat shock. In the meantime, the *Venus* expression cassette was amplified from the Venus expression plasmid (pcDNA3.1-Venus) using the primers Venus cassette F and Venus cassette R. Then, the purified PCR product (100 ng) was introduced into the cells to replace the *galK-Kn* cassettes. After incubation for 4.5 h, the cells were washed twice with M9 medium and plated onto M63 minimum medium plates supplemented with glycerol, leucine, biotin, 2-deoxygalactose, and CP. The bacterial colonies were screened, and a single positive colony was selected. The resultant BAC was named pBAC-CAdV2-ΔE1-Venus.

### 2.6. Propagation of the E1 Deletion Mutant of CAdV2

To construct the E1 expression plasmid, the *E1* region of CAdV2 (as described in a previous study [9]) was amplified by PCR and then cloned into HindIII–XhoI-digested pKS336 (GenBank accession no. AF403737) to generate pKS-CAdV2-E1. Then, this resultant plasmid was linearized through BsaI digestion and introduced into MDCK cells using polyethylenimine. At 24 h post-transfection, 15 µg/mL of blasticidin was added. After 4–5 days of incubation, the cells were subcultured for colony formation. These clones were screened and a single clone, named MDCK-CAdV2-E1-26, was chosen for use in this study. The expression of canine glyceraldehyde 3-phosphate dehydrogenase (*cGAPDH*), *E1A*, and *E1B* was confirmed by reverse transcription (RT)-PCR using the primers cGAPDH F and cGAPDH R, CAdV2-E1A F and CAdV2-E1A R, and CAdV2-E1B F and CAdV2-E1B R, respectively.

To generate recombinant CAdVs, pBAC-CAdV2-ΔE1-Venus was first digested with PvuI, following which the linearized viral genomic DNA was transfected into MDCK-CAdV2-E1-26 cells using polyethylenimine. At 5–6 days post-transfection, the medium and the cells were frozen and thawed three times, and the supernatants obtained from centrifugation of the cell cultures were collected and used to infect the MDCK-CAdV2-E1-26 cells. Once 50–80% of the cells showed cytopathic effects, the culture supernatants were collected and stored at −80 °C.

### 2.7. Growth Kinetics of the Recombinant Viruses in Cell Culture

MDCK cells were infected with CAdV-WTs or CAdV-rWTs at a multiplicity of infection (MOI) of 0.01. After incubation at 37 °C for 1 h, the inocula were removed completely. Then, following a wash with DMEM supplemented with 1% FBS, the cells were maintained in DMEM supplemented with 1% FBS, and the culture supernatants were collected daily for up to 6 days post-infection (dpi). To measure the viral titers, plaque assays were performed with MDCK cells. After virus absorption to the cells in 12-well plates at 37 °C for 1 h, the inocula were removed, and the cells were overlaid with Eagle’s minimal essential medium supplemented with 1% FBS and 0.8% agarose. At 4 dpi, the agarose was removed, and the plaques were fixed with methanol and stained with 0.1% crystal violet for counting the plaque numbers. To determine the growth properties of CAdV2-ΔE1-Venus, MDCK and MDCK-CAdV2-E1-26 cells were infected with CAdV2-rWT and CAdV2-ΔE1-Venus at an MOI of 0.2 and then further processed as described above. The viral titers (median tissue culture infectious dose (TCID_50_)/mL) were measured using MDCK-CAdV2-E1-26 cells. To authenticate that *E1* had been deleted in CAdV2-ΔE1-Venus after the viral propagation in MDCK cells, viral DNA was extracted from the culture supernatants of the infected cells and used as a template to amplify the *hexon*, *E1A*, and *E1B* gene regions using the primers CAdV2-hexon F and CAdV2-hexon R, CAdV2-E1A F and CAdV2-E1A R, and CAdV2-E1B F and CAdV2-E1B R, respectively.

### 2.8. Statistical Analysis

The data of the viral growth kinetics in MDCK and MDCK-CAdV2-E1-26 cells were analyzed using Student’s *t*-test, with two-tailed analysis, to determine the statistical significance of differences.

## 3. Results

### 3.1. Construction of Bacterial Artificial Chromosome Clones of CAdV1 and CAdV2

**Figure 1:**
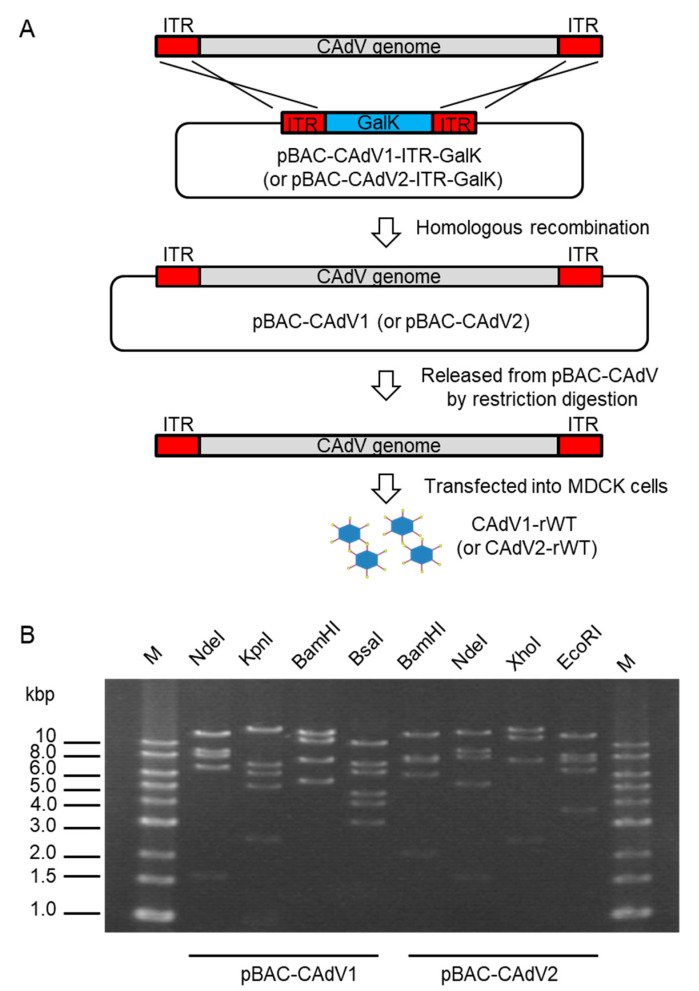
Cloning of canine adenovirus (CAdV) genomes into bacterial artificial chromosome (BAC) vectors. (**A**) CAdV1 (or CAdV2) DNA was introduced into SW102 cells harboring pBAC-CAdV1-ITR-GalK (or pBAC-CAdV2-ITR-GalK). The plasmid contains a galactokinase (*galK*) expression cassette flanked by approximately 50 bp of inverted terminal repeats (ITR) of the viral genome. After homologous recombination by Red recombinase, cells were selected using GalK, and pBAC-CAdV1 (or pBAC-CAdV2) was obtained. Then, this plasmid was digested with a restriction enzyme and transfected into Madin–Darby canine kidney (MDCK) cells, following which the recombinant virus CAdV1-rWT (or CAdV2-rWT) was rescued. (**B**) Restriction enzyme analysis of pBAC-CAdV1 and pBAC-CAdV2. pBAC-CAdV1 was digested with NdeI, KpnI, BamHI, and BsaI, whereas pBAC-CAdV2 was digested with BamHI, NdeI, XhoI, and EcoRI. The predicted molecular weights of the digested fragments of pBAC-CAdV1 are 13099, 8710, 8038, 6737, and 1603 bp for NdeI; 16319, 7077, 6210, 5110, 2490, and 981 bp for KpnI; 13573, 10982, 7620, 5549, and 463 bp for BamHI; and 10530, 7212, 6386, 4670, 4041, 3117, 825, 493, 471, 153, 147, and 142 bp for BsaI. The predicted molecular weights of the digested fragments of pBAC-CAdV2 are 13521, 7998, 7620, 6151, 2109, 851, and 722 bp for BamHI; 14820, 9059, 8127, 5363, and 1603 bp for NdeI; 16628, 12194, 7688, and 2462 bp for XhoI; and 13014, 8192, 7592, 6532, and 3642 bp for EcoRI. M: 1-kb DNA ladder marker.

To construct infectious BAC clones of CAdVs, we first determined the full-length sequences of the CAdV1 (strain D43) and CAdV2 (strain A2) genomes using next-generation sequencing, indicating 30,533 bp and 31,320 bp as the full lengths and mapping their identical gene arrangements to those of the reference viruses, respectively. Consequently, pBAC-CAdV1-ITR-GalK and pBAC-CAdV2-ITR-GalK were constructed, and the full-length viral genomes were then inserted into the BAC vector through homologous recombination with Red recombinase in SW102 cells. After the SW102 cell colonies had been screened by PCR, pBAC-CAdV1 and pBAC-CAdV2 were obtained (Figure 1A), and their proper constitutions were confirmed from their restriction enzyme digestion patterns (Figure 1B).

### 3.2. Generation and Growth Properties of Recombinant CAdV1 and CAdV2

**Figure 2:**
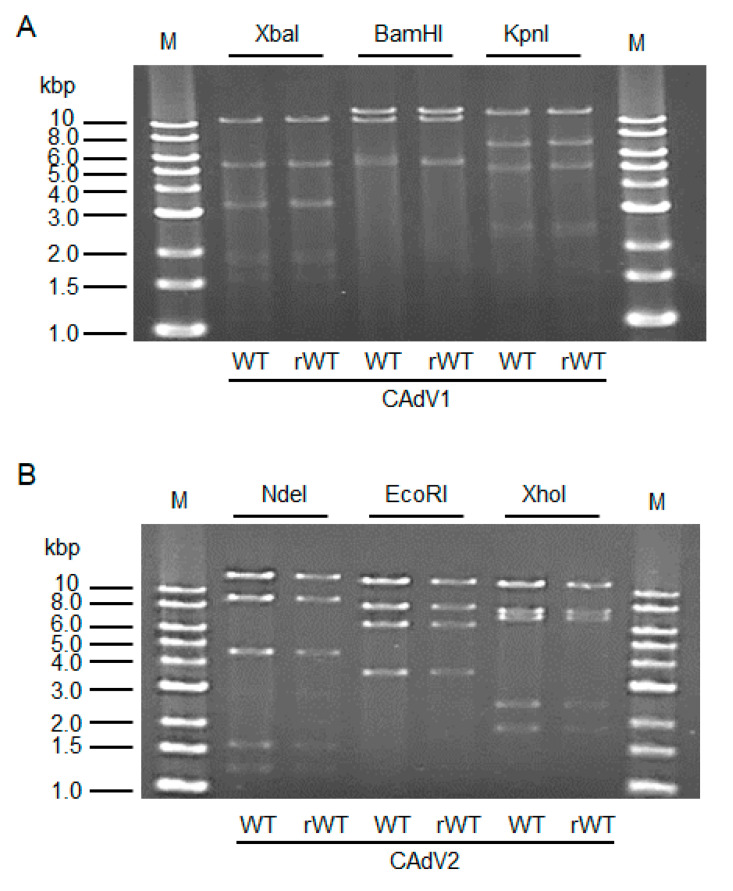
Restriction enzyme analysis of the genomes of wild-type (WT) and recombinant wild-type (rWT) viruses. (**A**) The genomes of CAdV1-WT and CAdV1-rWT were digested with XbaI, BamHI, and KpnI. The predicted molecular weights of the digested fragments of the viral genomes are 11250, 5551, 3383, 3301, 1955, 1834, 1587, 775, 519, 333, and 45 bp for XbaI; 13554, 10982, 5534, and 463 bp for BamHI; and 12573, 7077, 5110, 2490, 2302, and 981 bp for KpnI. M: 1-kb DNA ladder marker. (**B**) The genomes of CAdV2-WT and CAdV2-rWT were digested with NdeI, EcoRI, and XhoI. The predicted molecular weights of the digested fragments of the viral genomes are 14820, 9059, 4585, 1603, and 1253 bp for NdeI; 13014, 8192, 6504, and 3610 bp for EcoRI; and 12194, 7688, 7089, 2462, and 1887 bp for XhoI. M: 1-kb DNA ladder marker.

**Figure 3:**
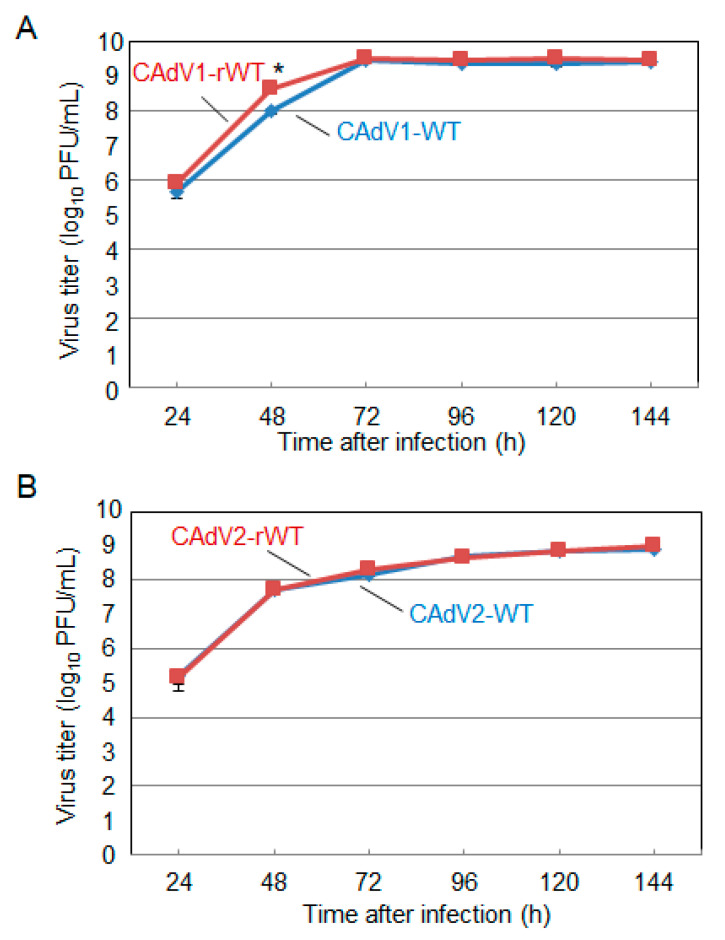
Growth kinetics of the wild-type (WT) and recombinant wild-type (rWT) viruses. MDCK cells were infected with (**A**) CAdV1-WT or CAdV1-rWT and (**B**) CAdV2-WT or CAdV2-rWT at a multiplicity of infection (MOI) of 0.01. The culture supernatants were collected at the indicated time points, and the viral titers were determined by plaque assay. The results were reported as the mean titer with standard deviations for three independent experiments. The asterisk indicates a significant difference (*p* < 0.05 by Student’s *t*-test).

The CAdV DNAs were released from pBAC-CAdV1 or pBAC-CAdV2 by restriction enzyme digestion and transfected into MDCK cells. Then, infectious viruses (CAdV-rWTs) showing clear cytopathic effects were obtained from the transfected cells, following one additional passage with MDCK cells. The restriction enzyme digestion patterns of the CAdV-rWT genomes were identical to those of the CAdV-WTs (Figure 2A,B), suggesting a lack of major DNA rearrangement in the recombinant viruses. Additionally, the growth properties of the CAdV-rWTs were similar to those of the CAdV-WTs in MDCK cells (Figure 3A,B). These data indicated that the recombinant viruses with WT phenotypes could be efficiently and easily rescued by pBAC-CAdV1 or pBAC-CAdV2 clones, verifying our successful establishment of a BAC-based reverse genetics system for CAdVs.

### 3.3. Generation of a Recombinant E1 Deletion Mutant of CAdV

The adenovirus *E1* region is divided into *E1A* and *E1B*. The *E1A* gene products transactivate the expression of other viral early genes and interact with cellular proteins, pushing the cell cycle into the S phase to support viral DNA replication [26]. The *E1B* gene encodes two major polypeptides, E1B 19K and E1B 55K; the former suppresses apoptosis, whereas the latter selectively exports viral late mRNAs from the host cell nucleus [27]. Since these genes are required for efficient virus replication, deletions of E1 or E1A will render most adenoviruses in the *Mastadenovirus* genus replication defective in normal cells, meaning that these E1-lacking viruses could be used as safe gene transfer vectors [28].

**Figure 4:**
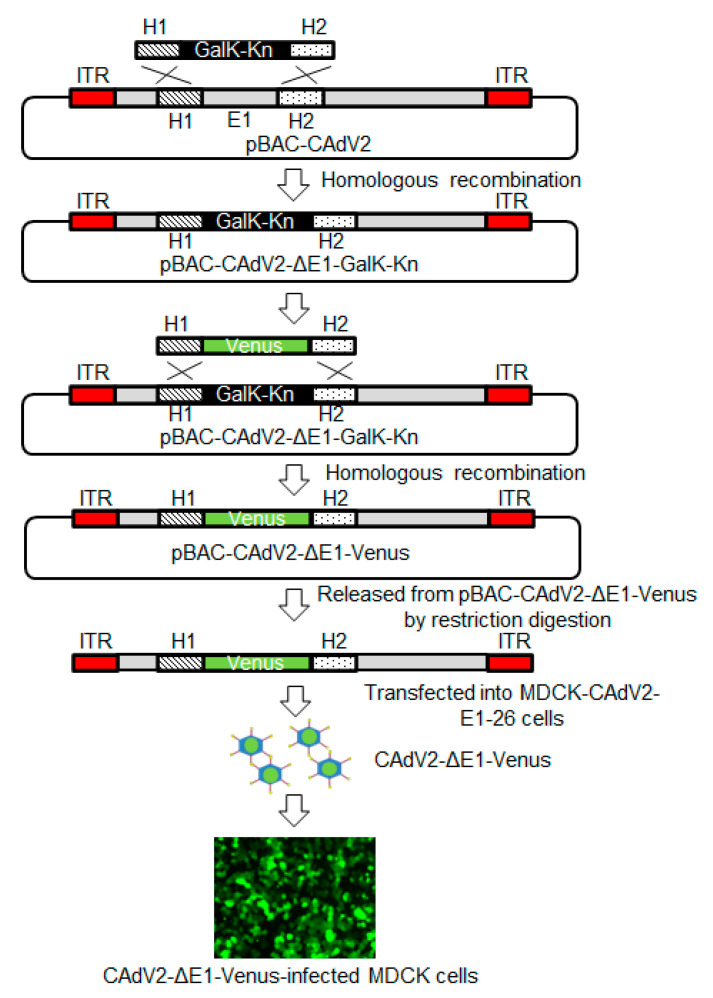
Generation of the E1-deleted CAdV2 mutant. The *E1* region of pBAC-CAdV2 was replaced with the *Venus* expression cassette. The *galK* and kanamycin resistance gene (*galK-Kn*) expression cassette, flanked by homologous region 1 (H1) and region 2 (H2), was generated by PCR and introduced into SW102 cells harboring pBAC-CAdV2. After homologous recombination, cells were selected by GalK and Kn, whereupon those harboring pBAC-CAdV2-ΔE1-GalK-Kn were obtained. The *Venus* expression cassette flanked by H1 and H2 was generated by PCR and introduced into the SW102 cells harboring pBAC-CAdV2-ΔE1-GalK-Kn. After homologous recombination, cells were selected by GalK, whereupon those harboring pBAC-CAdV2-ΔE1-Venus were obtained. CAdV2-ΔE1-Venus was rescued through the transfection of restriction enzyme-digested pBAC-CAdV2-ΔE1-Venus into MDCK-CAdV2-E1-26 cells. After the MDCK cells had been infected with CAdV2-ΔE1-Venus at an MOI of 0.4, Venus expression was observed by fluorescence microscopy. The image was taken at 24 h post-infection.

**Figure 5:**
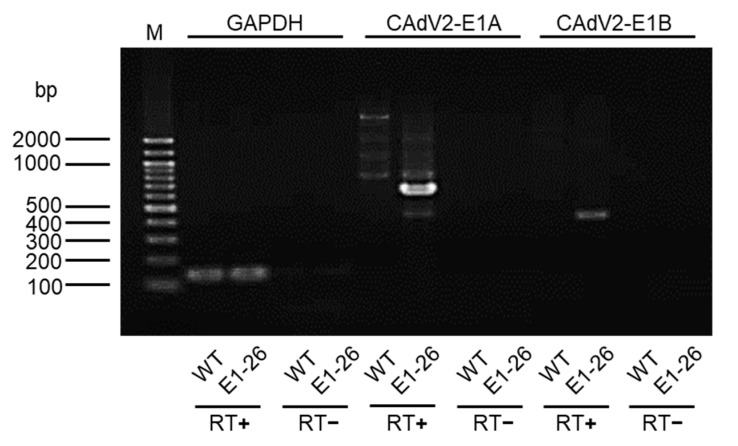
CAdV2 *E1* gene expression in MDCK-E1-26 cells. RNA was extracted from MDCK wild-type (WT) or MDCK-CAdV2-E1-26 cells (E1-26). RT-PCR was performed using primers specific for canine glyceraldehyde 3-phosphate dehydrogenase *GAPDH*, CAdV2-*E1A*, and CAdV2-*E1B*, with (+) or without (−) reverse transcriptase (RT). M: 100-bp DNA ladder marker.

To validate the utility of our BAC-based reverse genetics system, we used it to generate E1-deleted CAdV2. We prepared a mutant construct, pBAC-CAdV2-ΔE1-Venus, the *E1* region of which was replaced with a fluorescent *Venus* expression cassette (Figure 4). Additionally, to efficiently generate E1-deleted CAdV2, MDCK-CAdV2-E1-26 cells that could constitutively express CAdV2 E1 were prepared by transfecting MDCK cells with pKS-CAdV2-E1, followed by cell cloning with antibiotics. The expression of CAdV2 *E1A* and *E1B* in these cells was confirmed by RT-PCR (Figure 5). Then, we transfected MDCK-CAdV2-E1-26 cells with the CAdV2-ΔE1-Venus DNA that had been released from pBAC-CAdV2-ΔE1-Venus through restriction digestion, resulting in the generation of the infectious Venus-expressing virus CAdV2-ΔE1-Venus (Figure 4). The proper sequence around the genetically modified region was confirmed in CAdV2-ΔE1-Venus. These data suggested the usefulness of our BAC-based reverse genetics system for the generation of mutant viruses.

### 3.4. Growth Properties of the Recombinant E1 Deletion Mutants of CAdVs

**Figure 6:**
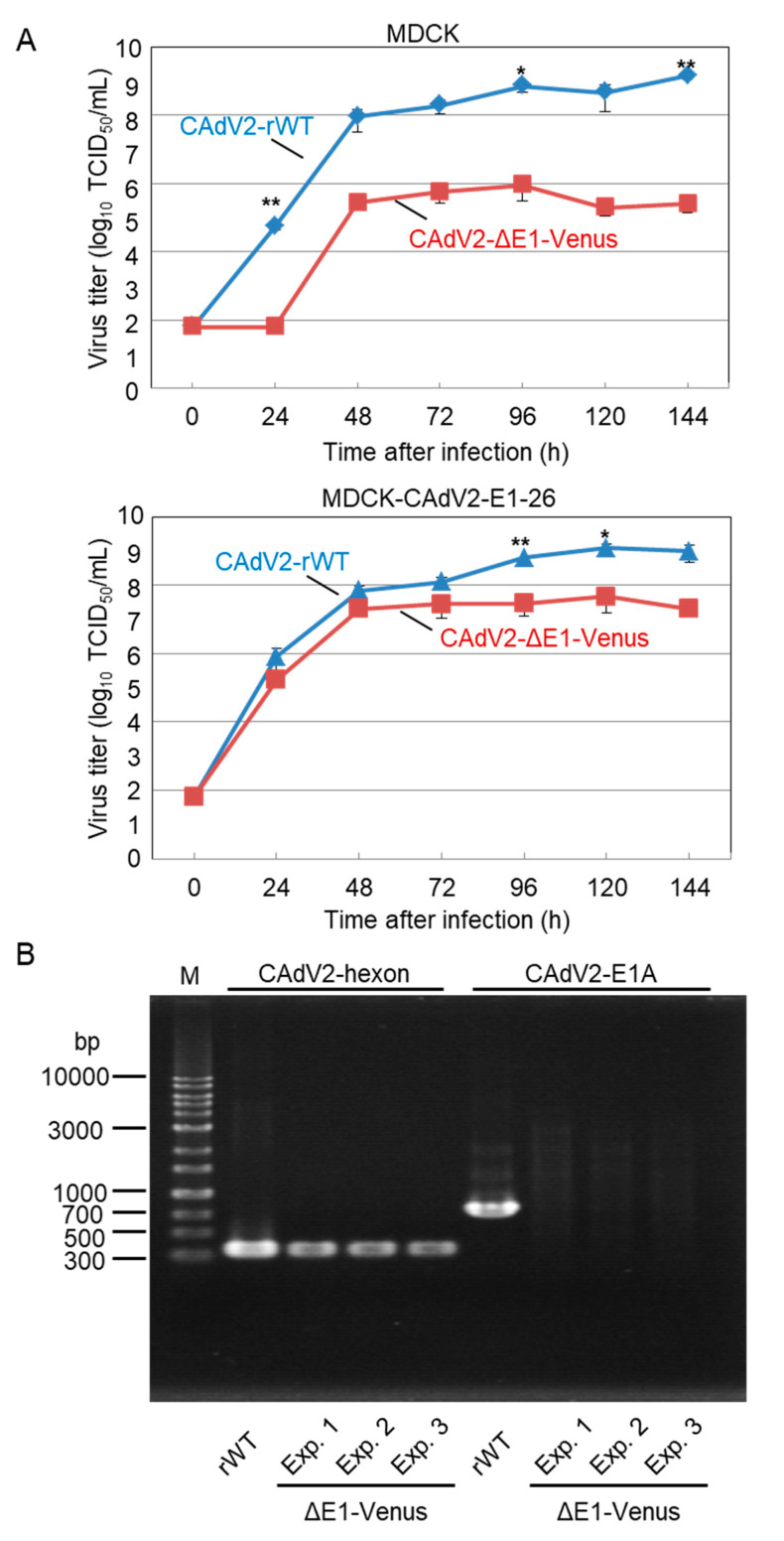
Characterization of CAdV2-ΔE1-Venus. (**A**) Growth kinetics of CAdV2-WT and CAdV2-ΔE1-Venus. MDCK cells and MDCK-CAdV2-E1-26 cells were infected with CAdV2-WT or CAdV2-ΔE1-Venus at an MOI of 0.2. The culture supernatants were collected at the indicated time points, and the viral titers (TCID_50_/mL) were determined in MDCK-CAdV2-E1-26 cells. The results are reported as the mean titer with standard deviations for three independent experiments. The asterisks indicate significant differences (* *p* < 0.05; ** *p* < 0.01 by Student’s *t*-test). (**B**) Analysis of the CAdV2-rWT and CAdV2-ΔE1-Venus genomes. CAdV2-ΔE1-Venus was generated by three independent experiments (Exp. 1, 2, and 3). Viral DNA was extracted from CAdV2-rWT and CAdV2-ΔE1-Venus propagated in MDCK cells. PCR was performed using primers specific for the CAdV2 *hexon* and *E1A* genes. M: 1-kb DNA ladder marker.

Using our generated E1-deleted mutant, we investigated whether CAdV2 requires its *E1* gene products for efficient replication in cells in the same way that other adenoviruses of genus *Mastadenovirus* do. A comparison of the growth properties of CAdV2-rWT and CAdV2-ΔE1-Venus in MDCK cells revealed that viral titers of CAdV2-ΔE1-Venus were more than 2-log lower than those of CAdV2-rWT at any time point (Figure 6A). This reduction in CAdV2-ΔE1-Venus growth was corrected, albeit incompletely, in MDCK-CAdV2-E1-26 cells (Figure 6A).

To rule out the possibility that the replication of CAdV2-ΔE1-Venus in MDCK cells may have been due to a regain of the *E1* gene through homologous recombination in overlapping sequences between CAdV2-ΔE1-Venus DNA and adenovirus-derived DNA in MDCK-CAdV2-E1-26 cells [29,30], the DNA of CAdV2-ΔE1-Venus propagated in MDCK cells was extracted and examined for its *E1* gene content by PCR. Although the *hexon* gene was detected, the *E1A* and *E1B* genes were not detected in the CAdV2-ΔE1-Venus DNA (Figure 6B). Taken together, these data indicated that the *E1* gene products were not essential for the replication of CAdV2 in MDCK cells but were required for its efficient growth.

## 4. Discussion

The BAC-based methodology, designed for the robust cloning and propagation of large and unstable DNA fragments in *Escherichia coli*, has been utilized in the genetics of DNA viruses with large genome sizes, such as herpesviruses [31]. This method has been adapted for the manipulation of human or simian adenoviral genes, suggesting its applicability for adenovirus genetics [32,33]. In this study, we established a BAC-based reverse genetics system for CAdVs.

To approach basic studies on CAdVs (e.g., on their pathogenesis) as well as practical studies (e.g., on CAdV recombinant vaccines and CAdV-based vectors), a reverse genetics system is an essential tool whereby the intended gene manipulation can be introduced into the viral genome. Conventional reverse genetics systems for CAdVs are based on methods that include in vitro ligation, homologous recombination in mammalian cells, and homologous recombination in *E. coli* strain BJ5183. These methods require a long time and a great deal of effort to robustly generate infectious clones and mutant viruses, because of the instability of the viral DNA in the plasmids as well and the necessity for a unique restriction enzyme site or long homology arms to modify the genome [9,13,14,15,20]. To circumvent such bottlenecks, in this study, we applied the BAC cloning methodology to the reverse genetics system for CAdVs, requiring only short 50-bp homology arms [23], and successfully generated infectious BAC clones of CAdV1 and CAdV2 with a WT phenotype. Additionally, by using the system to generate an E1-deleted mutant of CAdV2, we showed that a genetic modification could be easily made through BAC recombineering. Taken together, our established BAC-based reverse genetics system is a simple and efficient method for the generation of recombinant CAdVs and is superior to the conventional methods.

As prospective approaches, the E1-deleted CAdV2 mutant may be used as a backbone to construct recombinant CAdV vaccines, gene transfer vectors, or oncolytic viruses [9,28,34]. Interestingly, this CAdV2 mutant was able to replicate in MDCK cells, albeit less efficiently than the WT virus. The requirement of *E1* genes for adenovirus replication in cells depends on the *Mastadenovirus* species; for example, E1-deleted HAdV5 is replication defective in normal cells and has been used as a safe vector for a long time [35], and E1A-deleted mouse adenovirus 1 can grow to a similar level as the WT virus in vitro [36]. Although we did not test other cell lines aside from MDCK cells in this study, previous studies have indicated that E1-like factors are expressed in some cell lines, supporting the replication of E1-deleted HAdV5 [37,38,39]. It would be interesting to investigate whether MDCK cells express E1-like factors that support the replication of E1-deleted CAdV2.

Our observation that the E1-deleted CAdV2 mutant replicated less efficiently than the WT virus in MDCK cells provides a possibility that this mutant may be attenuated in vivo, suggesting its potential as a live vaccine against CAdV infections. Therefore, this mutant-based recombinant constructs containing protective antigens of other pathogens such as rabies virus, canine distemper virus, canine influenza virus, etc., can be applied to multivalent vaccine strategy for canid animals, as approached in previous studies [16,40,41].

In conclusion, we have established BAC-based reverse genetics systems for CAdVs, through which gene manipulations could be efficiently, easily, and robustly performed. These systems could be useful and powerful tools for both basic and advanced practical studies on CAdVs.

## Supplementary Materials

The following is available online at https://www.mdpi.com/1999-4915/12/7/767/s1; Table S1: List of primers used in this study.
