# Supplementary material for: Establishment of a Simple and Efficient Reverse Genetics System for Canine Adenoviruses Using Bacterial Artificial Chromosomes
Source: Viruses. 2020 Jul 16;12(7):767. doi: 10.3390/v12070767 (PMC7412426; doi:10.3390/v12070767)
Supplement: Supplementary file 1 [file viruses-12-00767-s001.pdf]

Supplementary Information

Establishment of a simple and efficient reverse genetics system for canine adenoviruses using bacterial artificial chromosomes

Hiromichi Matsugo, Tomoya Kobayashi-Kitamura, Haruhiko Kamiki, Hiroho Ishida, Akiko Takenaka-Uema,

Shin Murakami and Taisuke Horimoto

Table S1. Primers used in this study

Primer	Sequence (5′ – 3′)	Sequence capitalized
pUC19-GalK F	gacgTCTAGAcctgttgacaattaatcatc	restriction enzyme site
Kn-Galk R	ccatcttgttcaatcatGTACAGCCTCCTtcagcactgtcctgctccttg	Shine-Dalgarno (SD) sequence
GalK Kn F	AGGAGGCTGTACatgattgaacaagatggattg	Shine-Dalgarno (SD) sequence
pUC19-Kn R	ggtcAAGCTTtcagaagaactcgtcaagaag	restriction enzyme site
CAdV1-ITR-GalK F1	GGACAAAGAGGTGTGGCCTAAATGTTGTTTTTcctgttgacaattaatcatcg	inverted terminal repeats (ITR) sequence
CAdV1-ITR-GalK R1	GGACAAAGAGGTGTGGCCTAAATGTTGTTTTTtcagcactgtcctgctccttg	inverted terminal repeats (ITR) sequence
CAdV2-ITR-GalK F1	GGACAAAGAGGTGTGGCTTAAATTTGGGCGTTcctgttgacaattaatcatcg	inverted terminal repeats (ITR) sequence
CAdV2-ITR-GalK R1	GGACAAAGAGGTGTGGCTTAAATTTGGGCGTTtcagcactgtcctgctccttg	inverted terminal repeats (ITR) sequence
CAdV1-ITR FR2	atctGGATCCTTAATTAACGATCGcatcatcaataatatacaggacaaagaggtgtggcctaaatgttg	restriction enzyme sites
CAdV2-ITR FR2	atctGGATCCGCGGCCGCGATCGcatcatcaataatatacaggacaaagaggtgtggcttaaatttgg	restriction enzyme sites
SL1	cagtccagttacgctggagtc	
SMART BAC 159R	aacctcttacgtgccgatcag	
CAdV1 259R	gctccaaggctagaacaataaacc	
CAdV2 300R	acattaggcgtggaggaactg	
CAdV1 30090F	gtctgtgagctccagatgag	
CAdV2 30880F	agaggagagctatgaggatg	
CAdV2-E1-GalK-Kn F	GGTTTTTCGCCACGCCTTTGGGTTAATTTTATTTCCCTATACGCGGCCTTcctgttgacaattaatcatcg	homologous sequence
CAdV2-E1-GalK-Kn R	ATTCTCTGCACAACCCCCCGAGGGGCTGCAATTACACACATTGACAAAGTtcagaagaactcgtcaagaagg	homologous sequence
Venus cassette F	ATTCTCTGCACAACCCCCCGAGGGGCTGCAATTACACACATTGACAAAGTgacattgattattgactag	homologous sequence
Venus cassette R	GGTTTTTCGCCACGCCTTTGGGTTAATTTTATTTCCCTATACGCGGCCTTccatagagcccaccgcatcc	homologous sequence
cGAPDH F	cgatggtgaaggtcggagtg	
cGAPDH R	actggaacatgtacaccatgtagtt	
CAdV2-E1A F	agagctactggaagagtggc	
CAdV2-E1A R	ttctgtttgctcaggctctg	
CAdV2-E1B F	cactttagaaatgcccagag	
CAdV2-E1B R	ctttcactaatgcaacacac	
CAdV2-hexon F	tacatgaatgttagattacc	
CAdV2-hexon R	ggttgatgctctgaatgttg	
